# Potato Periderm Development and Tuber Skin Quality

**DOI:** 10.3390/plants11162099

**Published:** 2022-08-12

**Authors:** Pawan Kumar, Idit Ginzberg

**Affiliations:** Institute of Plant Sciences, Agricultural Research Organization, Volcani Institute, 68 HaMacabim Road, P.O. Box 15159, Rishon LeZion 7505101, Israel

**Keywords:** cork, phellem, phellogen, russeting, *Solanum tuberosum*, tuber skin

## Abstract

The periderm is a corky tissue that replaces the epidermis when the latter is damaged, and is critical for preventing pathogen invasion and water loss. The periderm is formed through the meristematic activity of phellogen cells (cork cambium). The potato skin (phellem cells) composes the outer layers of the tuber periderm and is a model for studying cork development. Early in tuber development and following tuber expansion, the phellogen becomes active and produces the skin. New skin layers are continuously added by division of the phellogen cells until tuber maturation. Some physiological disorders of the potato tuber are related to abnormal development of the skin, including skinning injuries and russeting of smooth-skinned potatoes. Thus, characterizing the potato periderm contributes to modeling cork development in plants and helps to resolve critical agricultural problems. Here, we summarize the data available on potato periderm formation, highlighting tissue characteristics rather than the suberization processes.

## 1. The Plant Periderm

The periderm, or cork, is a protective tissue of secondary origin. Its defensive characteristics are mainly due to the deposition of suberin polyester on the walls of its outer cell layers. The suberin consists of aromatic and aliphatic domains crosslinked by glycerol, and is localized between the primary wall and the plasmalemma [1]. The suberized cells are filled with air and, therefore, provide thermal insulation, the suberized walls prevent invasion by microorganisms (mechanically and chemically), and wax deposits that are embedded in the suberin material prevent desiccation of internal tissues [1,2].

The periderm occurs in several plants and plant organs: in the skin of underground organs such as potato and sweet potato; the cork of woody species due to increased thickness via secondary growth; the lenticels; the wound-healing periderm [3]; the russeting of fruit peels of, e.g., apple, pear, and kiwifruit; the netted rind of reticulated fruit [4,5,6,7], etc. A review on periderm evolution and ontogenesis has been recently published [8].

The periderm is made up of three types of cells [9]. The outer layers are composed of suberized phellem cells. The inner layers, the phelloderm, are made up of parenchyma-like cells. Between the phellem and the phelloderm is a meristematic layer of secondary origin called the phellogen or cork cambium. Outward cell divisions of the phellogen form the phellem and inward divisions form the phelloderm.

## 2. Native Periderm of Potato

**Figure 1:**
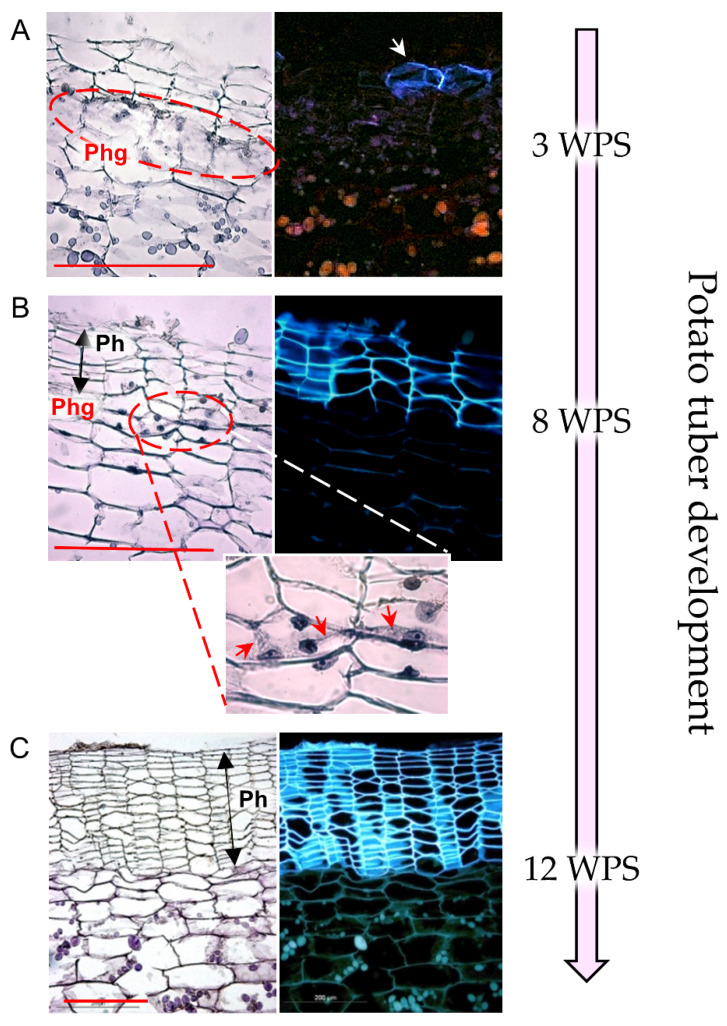
Developmental stages of potato native periderm. Potato cv. Desirée plants were grown in pots (20 L) filled with perlite in a greenhouse under natural Israeli winter conditions (average temperature range of 10–18 °C). Tubers were collected at three time points during tuber development—3, 8, and 12 weeks post-sprouting (WPS) of seed tubers. Tissue samples were taken from their surface, fixed in FAA, dehydrated in an ethanol-xylene series, and embedded in paraplast. Cross-sections were stained with hematoxylin and viewed under a light microscope (left panel) and a UV microscope (right panel, black background), to examine tissue morphology and cell nuclei and the autofluorescence of suberized cell walls, respectively. (**A**) Periderm initiation—subepidermal cells undergo dedifferentiation to form phellogen (Phg) initials (encircled) that sequentially produce the phellem cells (white arrow). (**B**) Immature periderm development—the phellogen remains active and adds more phellem (Ph) cells to the expanding tuber. The close-up image (X2.5 magnification) displays the dividing phellogen cells, showing cell plates between daughter cells (red arrows). The cell plate is prone to fracture, resulting in separation of the immature skin from the tuber surface. (**C**) Periderm maturation—following foliage removal or plant senescence, tuber expansion ceases, the phellogen cells stop dividing, and the skin-set process is induced. The phellogen layer is undetectable at the maturation stage. Note the characteristic morphology of the phellem: columns of flattened cells that autofluoresce under UV light. Scale bars: 200 µm. Images were taken from [13].

Following potato tuberization, the original epidermis is replaced by periderm tissue, with its outer suberized phellem cells comprising the tuber skin. The development of the potato periderm can be divided into three stages [10] (Figure 1): (1) periderm initiation—cambial phellogen is formed through dedifferentiation of subepidermal cells, and sequentially, skin formation is initiated; (2) immature periderm development—active phellogen adds more skin layers to the expanding tuber; the dividing phellogen is labile and prone to fracture, resulting in separation of the skin from the underlying tuber flesh, and resulting in the costly agricultural problem of skinning injuries [11,12]; (3) periderm maturation—when the tuber ceases to expand at the end of the growing period, no new skin cells are required and the phellogen becomes inactive. As a result, skin layers adhere strongly to the tuber flesh in a process known as skin set.

In addition to the protective characteristic of the suberized phellem, the immature skin of potato is enriched with proteins involved in defense responses to biotic and abiotic stresses [13]. Similarly, the parenchyma-like phelloderm is enriched with defensive secondary metabolites, such as anthocyanins and the toxic steroidal glycoalkaloids [14].

A study comparing potato skin and tuber-flesh transcriptomes, followed by functional analysis of genes that were highly and differentially expressed in the skin, demonstrated genes involved in developmental processes such as cell division, cell differentiation, morphogenesis, secondary cell wall formation (lignification and suberization), and stress-related activities [15,16]. Among these genes, several transcription regulators were identified that may play roles in skin initiation and maturation [15]. These included the homeobox-leucine zipper protein, *StHAT3-like* gene (Sotub01g029660), which is involved in controlling embryonic apical patterning and meristem function [17]; the *NUCLEAR TRANSCRIPTION FACTOR Y SUBUNIT B-3*, *StHAP3* (Sotub06g007420), which regulates embryo identity and development [18]; the MYB family *REGULATOR OF AXILLARY MERISTEMS 2,* *StRAX2-like* gene (Sotub09g006290), which controls a very early step in axillary meristem initiation [19]; the NAC (NAM/ATAF1/2/CUC)-domain protein, *StNAC83* (Sotub07g026930), which regulates shoot apical meristem formation [20]; the *RELATED TO APETALA AP2-LIKE*, *StRAP2-7-like* gene (Sotub11g029540), an ethylene-responsive transcription factor that is involved in the establishment of floral meristem identity [21]; *NO APICAL MERISTEM 4-LIKE, NAM4* (Sotub05g028670), and the transcription factor *TCP* (Sotub09g007560), which regulate the expression of flavonoid/anthocyanin-biosynthesis genes in response to the stress of intense light [22,23] and may be related to the accumulation of pigment in the red skin of cv. Desirée tubers.

## 3. Wound Periderm of Potato

**Figure 2:**
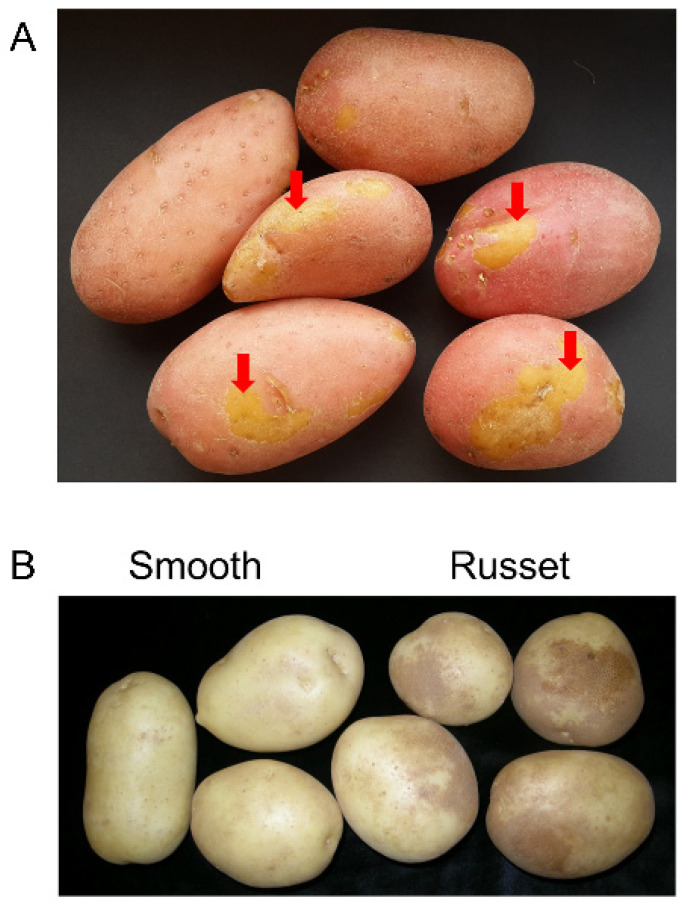
(**A**) Injuries of tuber surface healed by wound periderm that is devoid of the red pigmentation (red arrows). (**B**) Physiological russeting of smooth skin cultivar exhibiting dark brown patches of protruding rough skin tissue (right), compared to healthy-looking smooth skin tubers (left). Images were taken from [24] and [36], respectively.

Potato tuber skin is often damaged during mechanical harvest or due to an incomplete skin set. These skinning injuries are healed by the development of a wound periderm. Native and wound periderms are similar in terms of tissue origin, structure, and morphology, but differ in their maturation process and in the composition of unesterified pectin [12] and anthocyanin (in colored cultivars [24]) (Figure 2A). In addition, the suberin of the wound periderm is enriched in wax alkyl ferulates and is more permeable to water [25]. Recently, regulators of the wound-suberization process in tubers were identified—*StMYB74* and *StMYB102*, and polymorphisms in the latter were suggested to influence cultivar-specific wound-suberization capacity [26]. In potato, periderm development can be studied by inducing the formation of a wound periderm via removal of the tuber native skin or slicing the tuber flesh, and allowing the exposed tissue to heal in the dark. Within 1–3 days, wounding induces the formation of a closing layer, in which the walls of the exposed tuber parenchyma cells undergo lignification/suberization [10]. On day 3, phellogen initials become noticeable and columns of new phellem cells can be clearly seen below the closing layer [10,27,28,29]. From day 4 on, the newly formed phellem undergoes suberization from the outside layers inward, and on day 8, the suberized phellem layers become flattened and compacted, indicating maturation of the wound periderm [10].

A transient increase in auxin and lipid hydroxyperoxide levels at 20 to 30 min after wounding was suggested to initiate the cytological events that lead to wound periderm formation [30]. A mitotic activity of cells is followed to produce the periderm, starting 120 min post-wounding [30], parallel to the expression of cell-cycle genes [29], and an increase in polymerized actin and microfilament bundles in cells at the wounding site [31]. Further studies on hormonal regulation of wound periderm formation showed that abscisic acid, ethylene, and jasmonic acid levels increase transiently shortly after wounding and prior to periderm initiation [32,33,34,35].

Postharvest conditions of wounded tubers influence their capacity to heal. The effect of temperature, oxygen (O_2_) concentration, and relative humidity (RH) has been extensively studied in the context of the agricultural practice of curing skinning injuries of newly harvested potato tubers [27]. Wound-induced periderm formation occurred most rapidly at around 20–25 °C, was delayed at lower temperatures (10–15 °C), and was inhibited at temperatures as high as 35 °C [37,38]. The combination of O_2_ and temperature indicated inhibition at an O_2_ concentration of 1% or lower when stored at 15 °C or above [39]. In general, different combinations of temperature, O_2_, and RH should be adjusted according to the physiological state of the tubers [40] and local practices.

In this context, the timing of wound periderm initiation and development is critical for sealing the exposed internal tissues, to prevent pathogen invasion and water loss. Recently, brassinosteroid was shown to accelerate wound healing [41]. This steroidal hormone plays a positive role in the accumulation of lignin and polyphenolic suberin at the wound, reducing tuber weight loss and increasing disease resistance. The effect of brassinosteroid is achieved via upregulation of *PHENYLALANINE AMMONIA LYASE* (*PAL*), *4-COUMALATE:COA LIGASE* (*4-CL*), and *CINNAMYL ALCOHOL DEHYDROGENASE* (*CAD*) expression and related enzyme activities in phenylpropanoid metabolism, promoting the synthesis of lignin precursors and phenolic acids, mainly caffeic acid, sinapic acid, and cinnamyl alcohol [41]. In addition, increased production of O_2_^−^ and H_2_O_2_ following induction of NADPH oxidase promoted oxidative crosslinking of the phenolic acids and lignin precursors to form the polyphenolic domain of suberin [41].

## 4. Potato Skin Russeting

Impaired skin development that results in russeting of smooth-skinned varieties (Figure 2B) is often associated with suboptimal growth conditions [42]. This physiological skin disorder is not caused by pathogens [36]. Russeting may be a genetic trait, such as in the well-known US variety Russet Burbank where it is a desired character [43].

Tubers with russet skin have a thicker layer of phellem than smooth-skinned potatoes [36,42,44,45]. The buildup of phellem cell layers can result from increased activity of the phellogen as a result of, for example, high soil temperature [10,42] or strong adhesion between neighboring phellem cells so that they are not sloughed off during tuber development [36]. This may be due to increased suberization [45] or increased levels of pectin and hemicellulose [44]. Nevertheless, as the tuber expands during development, the thick skin cracks, resulting in netted or russet skin. We do not know if or how the undesirable phenomenon of physiological russeting is related to the russeting trait. That is, are these two different russeting phenomena determined by the same set of genes that interact with each other and with the environment to give different roughness outcomes, or are they determined by two different sets of genes? The roughness of Russet Burbank skin is dependent on growth temperature [45] and the quantity and source of fertilizer [46], suggesting that genetic and physiological russeting may involve the same set of genes.

## 5. Chemical Composition of the Periderm

### 5.1. Suberin

Our knowledge of the biosynthesis pathway and assembly of suberin macromolecules is not complete. However, recent studies have provided new information on genes involved in suberin regulation. These are detailed in two reviews included in the present Special Issue [47,48], and are briefly discussed here in the context of potato.

The aromatic domain of potato suberin is composed of monolignols and hydroxycinnamic (ferulic) acids, and is covalently bound to the primary cell wall [49,50]. The aliphatic domain is a polyester that, upon transesterification, releases mainly C16–C28 α,ω-diacids and ω-hydroxyacids, with minor amounts of alkan-1-ols, alkanoic acids, and glycerol. The latter may be involved in crosslinking between the aromatic and aliphatic domains [51,52].

Several enzymatic activities and associated gene families have been identified in suberin biosynthesis. β-Ketoacyl CoA synthases (KCSs) are involved in fatty-acid elongation, and in potato, StKCS6 catalyzes suberin and wax compounds with chain lengths of C28 and longer [53]. Cytochrome P450 (CYP) oxygenases play a crucial role in the end- and mid-chain oxidation steps, starting from monofunctional fatty acids and leading to the ω-hydroxyacids and α,ω-diacids [54]. In potato, CYP86A33 was shown to promote the ω-hydroxylation step, and its silencing led to a periderm with a 60% decrease in its aliphatic suberin load, mainly due to reduced levels of C18:1 ω-hydroxyacid and α,ω-diacid monomers (by about 70% and 90%, respectively) compared to the wild type, and increased periderm permeability [55]. Acyltransferases promote the esterification of suberin acids to glycerol and the esterification of ω-hydroxyacids to ferulic acid [56], and the potato glycerol phosphate acyltransferase-encoding *GPAT3* was found to be highly and differentially expressed at the phellogen-initiation stage [10].

The incorporation of ferulate into potato suberin is catalyzed by feruloyl transferase (FHT; [57]). It has been suggested that the product ω-hydroxyacid ferulate may be the preferred structure for linking the suberin aliphatic polyester to the neighboring aromatics [51]. Phenylpropanoid precursors and the activity of anionic peroxidase, which favored feruloyl substrates, may be involved in the synthesis of the aromatic domain [58,59].

Suberin also contains fatty-acid derivatives such as C18–C22 primary fatty alcohols that are catalyzed by fatty acyl-CoA reductases (FARs) [60]. In potato skin, *FAR3* was highly expressed in the developmental stages of primary phellem formation and was observed in the inner layers of the skin phellem; it was downregulated upon skin maturation [15].

Finally, ATP-binding cassette G-transporters (ABCGs) were shown to be required for the synthesis of an effective suberin barrier in roots [61], and *ABCG6* and *ABCG19* were found to be expressed in the potato phellogen transcriptome [10].

In addition, two orthologs of a protein family that mediates Casparian strip formation—CASP—in the suberized endodermis of Arabidopsis roots, *StCASP1B2* and *StCASP1*, were identified in potato skin, along with *StCYP86A22* and *StPOD72*, whose sequences suggest that they may be closely related to known suberin-related genes [15].

### 5.2. Other Chemical Components

In addition to the insoluble protective suberin polymer that is embedded in the phellem cell walls, the potato tuber periderm can be a source of protective chemicals, such as antioxidant, antibacterial, and insecticidal compounds [62,63]. These molecules can be biosynthetic intermediates of the suberin polyester, or independent defense metabolites.

Nonpolar metabolites include nonpolar waxes, saturated and unsaturated fatty acids, saturated dicarboxylic acids, monoacylglycerols, 1-alkanols, n-alkanes, sterols, and polyphenolics [64]. Polar metabolites include quinic acid, phenolic amines, phenolic acids, flavonoid glycoalkaloids (solanine, chaconine, leptin, solanidine, solatriose, etc.), saponins, polyamines (putrescine, spermine, and spermidine derivatives), and methylprotodioscin and protodioscin [64].

Wound periderms may contain similar metabolites. Polar extracts include polyphenolic amines (kukoamine isomers, feruloyl putrescine, and its isomer), spermidine derivatives, feruloyl tyramine, ferulic acid, and caffeoyl putrescine [63]. Additional metabolites are flavonoid glycosides (kaempferol glycosides), phenolic acids, the steroidal glycoalkaloids, hydroxycinnamoyl putrescines, chlorogenic acid, ferulic acid, iso-chlorogenic acid, caffeic acid, and coniferyl alcohol [65].

Wound healing is also associated with oxidative stress, and free radical-scavenging activities have been demonstrated in periderm extract [63]. In both native and wound periderms, metabolite composition and level may be cultivar-dependent.

## 6. The Phellogen—A Key Player in Periderm Formation

Despite the importance of the corky periderm in plants, there is a lack of knowledge on the mechanism of phellogen cells activity with respect to their initiation, proliferative activity, and inactivation following the completion of periderm development. The potato periderm and the cork of *Quercus suber* are the accepted models for studying cork development. Periderm development from the pericycle of Arabidopsis root and hypocotyl has also been described [66].

To characterize phellogen activity, potato phellogen cells were isolated by laser-capture microdissection and their transcriptome was analyzed [10]. The phellogen is a lateral meristem. Accordingly, the potato phellogen transcriptome shared more genes with the vascular cambium than with stem or root apical meristems or the flower meristem, and had 68% shared genes with the transcriptome of oak bark (phellem and phellogen) [10,67]. These included genes that determine specific aspects of the stem cell niche and radial patterning. Furthermore, several genes that were shown to be expressed in the inner layers of the potato skin, where phellem cells are newly formed, were also found to be expressed in the vascular cambium [15].

Having the potato phellogen transcriptome enabled the identification of cellular functions that play a role in its cambial activity—cell division and differentiation, histone modification, chromatin remodeling, a high level of ribosomal proteins to support the extensive translation activity, and stress-associated genes—including heat-shock proteins that have been reported to play a role in cell-cycle regulation and meristem dedifferentiation [10]. The regulation of phellogen cell-division activity in the potato tuber was suggested to involve modulation of cytokinin homeostasis [10], similar to the regulation of phellogen in *Populus* [68]. High levels of vesicle trafficking activity required for the formation of a cell plate between two daughter cells (Figure 1B) were also seen in the transcriptome of the phellogen. The deposition of a new cell wall is controlled by a cytoskeletal array known as the phragmoplast [69]. The potato phellogen transcriptome includes 145 genes that are putatively related to cell-plate assembly, including genes that code for actin and myosin, as well as different aspects of vesicle trafficking, the cytoskeleton, and microtubule organization.

## 7. Phellogen Putative Role in Physiological Tuber Skin Blemishes

The potato phellogen is agriculturally important. Several physiological disorders of potato skin including incomplete skin set, skinning injuries, skin russeting, and loss of red pigmentation in wounded periderms may be related to impaired phellogen activity [11,24,42]. Phellogen activity can be divided into three stages: (i) initiation, when skin phellem initials are formed; (ii) proliferation, in which the skin is formed continuously and is at its immature stage, (iii) inactivation, inducing the skin maturation/skin-set process (Figure 1). Characterization of the phellogen-associated genes involved in these stages may contribute to the improved management of physiological potato skin blemishes.

Preparation of the potato phellogen transcriptome enabled the identification of genes with differential and high expression levels (1) during phellogen initiation and the early stages of skin formation and (2) following phellogen inactivation and the induction of skin maturation and skin-set processes. Moreover, the unique differential expression patterns were confirmed in both native and wound periderms [10].

The initiation of potato phellogen requires the transition of somatic cells (hypodermis or tuber parenchyma cells) to pluripotent stem cells (phellogen) that can produce different types of progeny (i.e., phellem and phelloderm). During the somatic-to-meristematic transition, cells have to dedifferentiate, activate their cell-division cycle, and reorganize their physiology, metabolism, and gene-expression patterns [70]. Accordingly, specific potato phellogen-activation genes included the histones *H2B* (Sotub03g016600), *H3* (Sotub10g009520), and *H4* (Sotub11g029670); the inducer of procambial activity *VASCULAR TISSUE SIZE* (*VAS,* Sotub01g040060); the lignin/suberin-related *CAFFEOYL COENZYME A O-METHYLTRANSFERASE 1* (*CCoAOMT1,* Sotub02g031720), *GDSL LIPASE- like* (*CFT,* Sotub01g036860), and *GLYCEROL-3-PHOSPHATE SN-2-ACYLTRANSFERASE 3* (*GPAT3,* Sotub01g032090) [10]—with main functions involving the chromatin remodeling and cell-wall synthesis processes that are required for the establishment of a dedifferentiation state. *CFT* and *GPAT3* may also provide precursors for the synthesis of suberin at a later stage.

The phellogen inactivation/skin maturation (skin set)-related genes included *PHD-FINGER FAMILY HOMEODOMAIN PROTEIN/HAT3.1* (*PHDZnP/HAT3,* Sotub01g044570), *ACTIN 7* (*ACT7,* Sotub03g020330), *NON-RECOGNITION-OF-BTH 4/MEDIATOR 15* (*BTH4/MED15,* Sotub04g009440), *PEROXIDASE 49-LIKE* (*POD,* Sotub02g027930), *PEROXISOMAL DEFECTIVE 3/COMATOSE* (*PED3/CTS,* Sotub04g020700), *PROTEIN KINASE* (*APK1/AtATH8,* Sotub04g014120), and *ENHANCER OF AG-4 PROTEIN 2* (*HGF3/HUA2,* Sotub02g005440), with putative functions in the aging meristem [10].

## 8. Future Perspectives—Epigenetic Regulation of Potato Skin Development and Quality

Epigenetic factors such as DNA methylation and histone modifications have been shown to play a role in phellogen and phellem formation of cork oak and to have altered patterns in high- vs. low-quality phellem/cork [71,72,73,74]. Cork of low quality was characterized by the reduced meristematic activity of the phellogen, having a thinner cork layer, and the upregulation of genes belonging to the flavonoid pathway instead of promoting lignification and suberization [74]. These distinctions may not be applied to potato; however, they suggest that skin quality may be defined by phellogen characteristics. The russet skin disorder that develops on smooth-skinned potato cultivars (Section 4, above) may be viewed as a low-quality cork [36].

Potato displays strong genotype-by-environment interaction effects, and epigenetic regulation is known to govern plant responses to unfavorable growth conditions. Generally, the periderm/cork is a protective tissue that responds to stress conditions [13,45]. For example, the potato periderm was shown to react to heat stress by enhancing the production and accumulation of skin layers to create a thick and russeted protective cover [42]. Although the stress response in potato skin has not yet been shown to be epigenetically regulated, this demonstrates the potential for epigenetic modulation of skin development under unfavorable growth conditions. Agronomical practices may also induce chromatin modifications that affect skin quality [75,76]. A preliminary study determining global methylation level in potato skin indicated a significant reduction in the percentage of 5-methylcytosine (5-mC) in maturing skin compared to early immature skin, and in russet compared to smooth skin (personal communication). Similarly, a reduced concentration of 5-mC was determined in cork of low compared to high quality [73].

The potato phellogen transcriptome included orthologs of genes involved in epigenetic modifications in cork oak, including chromatin-remodeling genes, DNA methyltransferases, histone methyltransferases, and histone deacetylases [10]. Epigenetic factors were shown to be involved in lignification/suberization pathways of phellem cell walls [77] and may be regulated by the coordinated activity of histone variant H1.3 and the transcription factor MYB1 [78], which are also found in the potato phellogen transcriptome. Gene-expression studies indicated differential expression of some chromatin modifiers, higher in russeted vs. smooth skin (personal communication).

Taken together, the data support the hypothesis that epigenetic modifications are involved in regulating potato skin development and quality.

## 9. Conclusions

Periderm development is an interesting topic involving some notable biological processes, such as dedifferentiation of the hypodermis or parenchyma cells into “stem cells” and meristematic phellogen activity. The latter gives rise to two different cell types, each with its own differentiation pathway—the phellem cells accumulate suberin and then die, whereas the parenchyma-like phelloderm accumulates secondary defense proteins and metabolites. This makes studying periderm development a challenge, especially when every element in the formation of the tissue has implications for tuber skin/cork quality and the respective agricultural product. The formation of potato native and wound periderms has been studied for many years, focusing mainly on the suberization of the phellem cell wall, a process that gives the periderm its main protective feature. We still lack information on the regulation of phellogen cell activation to form the tuber skin, and inactivation to initiate skin maturation (skin set), the latter corresponding to skinning injuries, or russeting disorders in smooth-skinned potato cultivars, among other physiological problems. We summarized the most up-to-date knowledge on the potato periderm. Parallel studies on periderm development in trees, Arabidopsis, and russeted fruit skin will provide additional information that may be relevant for potato tuber skin.

## Data Availability

Not applicable.
